# First-in-Human Application of Very High-Power Short-Duration RF Ablation for Refractory AVNRT: A Case Report

**DOI:** 10.3390/life15121834

**Published:** 2025-11-29

**Authors:** Milos Babic, Milosav Tomovic, Dejan Vukajlovic, Vasko Zugic, Aleksandra Grbovic, Masa Petrovic, Milovan Bojic, Aleksandra Nikolic

**Affiliations:** 1 Institute for Cardiovascular Diseases “Dedinje”, 11000 Belgrade, Serbia; 2 Academy of Sciences and Arts of the Republika Srpska, 71126 Banja Luka, Bosnia and Herzegovina; 3 Faculty of Medicine, University of Belgrade, 11000 Belgrade, Serbia

**Keywords:** atrioventricular nodal reentrant tachycardia, very high-power short-duration ablation, radiofrequency ablation, slow pathway modification, first-in-human

## Abstract

Background: Refractory atrioventricular nodal reentrant tachycardia (AVNRT) is a rare condition, but poses a clinical challenge after failed standard ablation. Very high-power, short-duration (vHPSD) radiofrequency (RF) ablation has not yet been explored in slow pathway ablation/modification. Case Summary: A 61-year-old woman with recurrent AVNRT despite two prior ablations and multiple antiarrhythmics underwent successful slow pathway ablation using a 90-watt, 4-s vHPSD protocol. CARTO-guided mapping localized the presumed slow pathway, followed by several 90 W applications. Transient second-degree AV block (Wenckebach type) occurred and resolved spontaneously. The patient remained arrhythmia-free at 6-month follow-up. Conclusions: This is the first reported clinical use of 90 W/4 s RF energy for AVNRT. The vHPSD approach may offer an effective alternative for patients with refractory AVNRT.

## 1. Introduction

Atrioventricular nodal reentrant tachycardia (AVNRT) is the most frequent type of paroxysmal supraventricular tachycardia and is typically treated with radiofrequency (RF) slow pathway ablation. Standard ablation protocols (20–50 W for 30–60 s) achieve high acute success rates; however, a subset of patients experience arrhythmia recurrence or fail multiple procedures [1,2]. Therefore, innovations in the technique of RF ablation of AVNRT remain clinically relevant [3]. During some procedures, attempts to achieve durable slow pathway modification are interrupted by the occurrence of transient atrioventricular (AV) block, prompting the use of alternative strategies such as cryoablation or a transseptal left atrial approach [4].

Very high-power short-duration (vHPSD) ablation has gained increasing use in the treatment of atrial fibrillation and ventricular arrhythmias, owing to its procedural efficiency and potentially favorable safety profile. However, to date, the application of very high-power ablation (90 W) for slow pathway modification in AVNRT has not been reported in human patients.

We present the first clinical case of successful treatment of refractory AVNRT using vHPSD ablation at 90 W for 4 s.

## 2. Case Description

A 61-year-old woman with a history of paroxysmal supraventricular tachycardia (SVT) since 2016 presented with frequent symptomatic episodes requiring multiple emergency department visits for intravenous antiarrhythmics. She had also experienced two ECG-confirmed episodes of atrial fibrillation.

Two prior RF ablation procedures had been performed: slow pathway ablation for AVNRT in February 2023, and combined AVNRT ablation with pulmonary vein isolation (PVI) in May 2023. In both procedures, a significant proportion of RF applications were prematurely interrupted due to the appearance of non-conducted atrial signals. Each procedure was concluded after achieving AV nodal modification (presence of a slow pathway zone with non-inducible tachycardia). Despite these interventions, the patient continued to experience weekly SVT episodes, refractory to escalating doses of antiarrhythmics.

Transthoracic echocardiography showed normal structure and function, with a borderline enlarged left atrium (41 mm).

In 2025, a third electrophysiology study was performed using the CARTO 3D electroanatomical mapping system (Biosense Webster). During basic atrial pacing at a cycle length of 700 ms, slow pathway conduction was confirmed (prolonged AH interval). An activation map was created to localize the slow pathway region, approximately 15 mm inferior to the His bundle signal. A second activation map was generated during tachycardia.

Radiofrequency applications were delivered using a QDOT catheter (Biosense Webster) at 90 W for 4 s per application. Multiple applications were delivered at the presumed slow pathway site rendering AVNRT non-inducible, although residual slow pathway conduction persisted.

**Figure 1:**
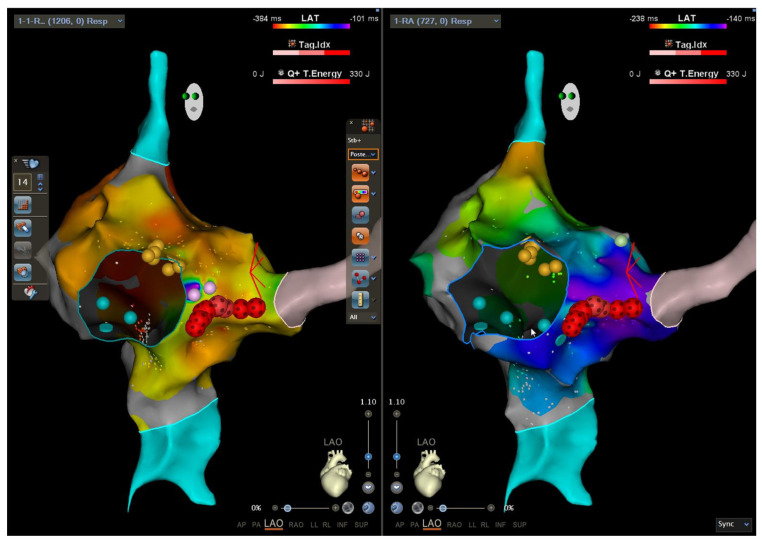
Three−dimensional electroanatomic mapping and ablation of the right atrium in the Left anterior oblique (LAO) showing the activation map during atrial pacing and conduction via slow pathway (**left side**) and during sinus activation (**right side**). The yellow dots depict the location of the His bundle as recorded by an electrode mapping catheter. The light blue structures represent superior and inferior vena cava. The grey structure represents the location of the coronary sinus with ostium indicated. The blue circle outlines the tricuspid annulus. The red-black tags were created by radiofrequency ablation utilizing the QDOT Catheter in the QMODE+ ablation mode (90 W/4 s).

**Figure 2:**
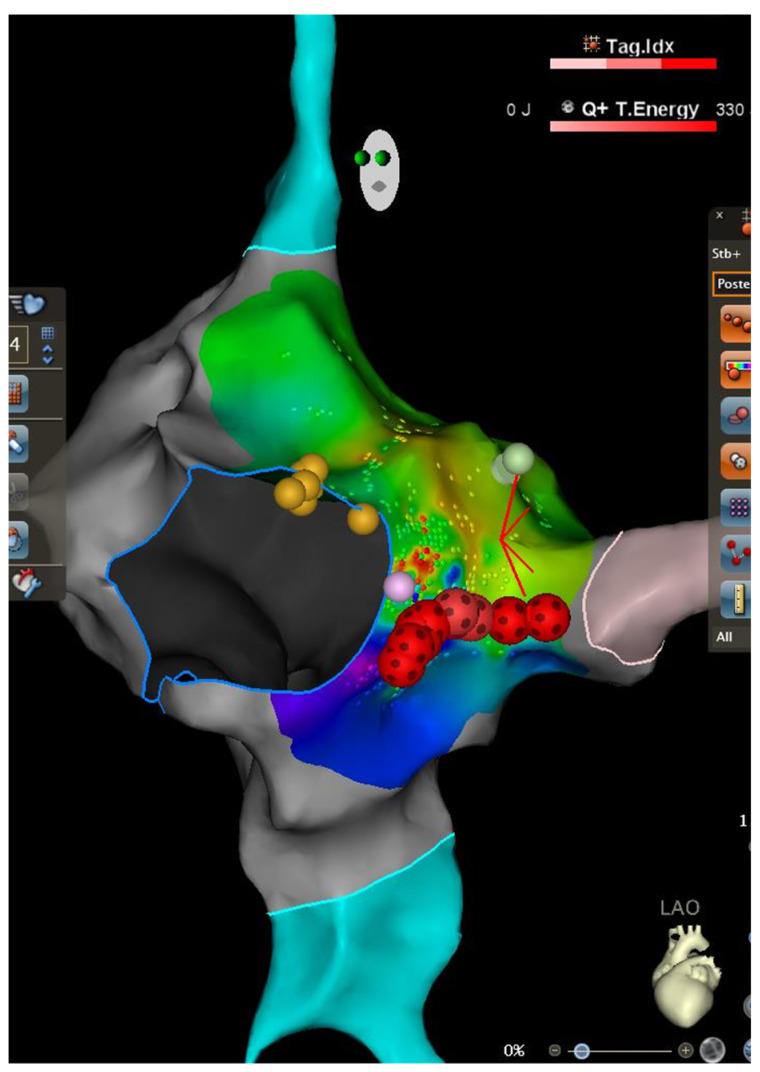
Three-dimensional electroanatomic mapping and ablation of the right atrium in the Left anterior oblique (LAO) showing the activation map of the right atrium during AVNRT. The yellow dots depict the location of the His bundle as recorded by an electrode mapping catheter. The light blue structures represent superior and inferior vena cava. The grey structure represents the location of the coronary sinus with ostium indicated. The blue circle outlines the tricuspid annulus. The red-black tags were created by radiofrequency ablation utilizing the QDOT Catheter in the QMODE+ ablation mode (90 W/4 s).

Two additional RF applications were then delivered near the coronary sinus ostium (Figure 1 and Figure 2). Immediately afterward, the patient developed transient second-degree atrioventricular block (Wenckebach pattern) with an atrial rate of 70 bpm, lasting several tens of minutes before spontaneous restoration of normal AV conduction.

In total, ten ablation points were applied, two of which were shorter than 4 s due to transient catheter instability. The average contact force was 10 g (range: 7–15 g), with an average temperature of 44.1 °C (range: 42–50 °C). The mean impedance drop achieved was 10 Ω, ranging from 5 Ω (during interrupted applications) to a maximum of 22 Ω.

Overall, eight full applications of 4 s each and two interrupted applications were performed, resulting in a total RF delivery time of 36 s. Given the anatomical specificity of the target zone and the ablation type, overlapping lesions were applied, maintaining a maximum inter-lesion distance of 4 mm.

A 24-h Holter ECG recorded the following day confirmed intact AV conduction with no recurrence of arrhythmia or AV block. The patient was discharged in sinus rhythm and remained asymptomatic, with no recurrence of AVNRT on follow-up at six months.

The patient was kept under observation the day after the procedure, during which a 24-h Holter ECG was performed. The monitoring confirmed intact atrioventricular conduction with no recurrence of arrhythmia or AV block. The patient was discharged in sinus rhythm on the second postoperative day and was advised to attend regular follow-up visits at her regional health center.

She was also contacted by telephone on two occasions to assess her clinical status. At the six-month follow-up, both clinical examination and ECG findings were normal. The patient remained asymptomatic, with no recurrence of AVNRT during the follow-up period.

## 3. Discussion

This case represents the first-in-human clinical use of a vHPSD ablation protocol (90 W for 4 s) for slow pathway modification in AVNRT.

AVNRT is the most frequent form of regular paroxysmal supraventricular tachycardia, and catheter ablation of the slow pathway is the established curative therapy. Both radiofrequency (RF) and cryothermal ablation techniques have demonstrated high efficacy and safety, with freedom from recurrence exceeding 90% in most series [5]. However, procedural failure and recurrences may still occur, particularly in patients with atypical septal anatomy, superiorly displaced slow pathways, or prior unsuccessful ablations.

In the present case, two previous right-sided RF ablations failed to achieve durable elimination of AVNRT. During the third intervention, alternative strategies were considered, including a left-sided ablation approach and cryothermal energy delivery. The final choice of technique was guided by a balance between procedural safety, efficacy, and institutional experience.

Left-sided ablation of AVNRT, typically via the mitral annulus or left septal region, has been described as a rescue option after failed right-sided procedures [6]. The rationale lies in the existence of left inferior or septal slow pathway inputs that may not be accessible from the right atrial side. Mapping studies have shown that, in a minority of patients, the earliest atrial activation during tachycardia can be recorded near the left posterior septum or along the mitral annulus. Although several case series have demonstrated successful AVNRT elimination from the left side, cumulative experience remains limited. A review summarizing approximately 80 cases reported acute success rates above 90%, with no permanent AV block, yet these findings originate from small, heterogeneous series in specialized centers [7]. In our institution, left-sided slow pathway ablation is rarely performed due to limited published evidence on long-term safety and the small number of suitable patients. Therefore, the potential procedural and anatomical risks were judged to outweigh the benefits.

Cryoablation represents another alternative. Its main advantage is the ability to perform cryomapping, allowing transient reversible freezing (typically −30 °C) to assess conduction effects before creating a permanent lesion (−70 °C). This provides a safety margin when ablating near the compact AV node or His bundle [8]. Meta-analytic evidence supports this safety benefit: in a pooled analysis of over 5600 patients, no permanent AV block occurred with cryoablation, compared with 0.7% in RF ablation [5]. Similarly, a long-term registry of 515 patients treated with cryoablation reported no late AV block over seven years, though recurrences occurred in approximately 11% of cases [9]. Thus, while cryoablation offers superior safety, its efficacy may be modestly reduced, particularly in redo procedures. Considering the patient’s prior ablation failures, we determined that cryothermal energy was less likely to yield a durable result.

Despite two previous unsuccessful right-sided procedures, we opted for a third attempt using a refined RF approach. This decision was supported by extensive safety data and improved lesion control through modern mapping technologies. The risk of complete AV block during right-sided RF ablation remains low when energy is delivered within the posteroinferior triangle of Koch. Failures typically result from an anatomically displaced slow pathway or inadequate lesion delivery due to suboptimal catheter stability. Integration of three-dimensional electroanatomical mapping and intracardiac echocardiography has improved both accuracy and safety, allowing more precise localization of the slow pathway conduction zone.

In conventional RF ablation, energy delivery of 30–50 W for 30–60 s creates lesions via resistive and conductive heating, producing deeper tissue injury that risks damaging nearby structures, particularly critical in AVNRT, given the proximity of the compact AV node. In contrast, vHPSD ablation delivers high power for short durations, producing wide and shallow lesions with minimal conductive heat spread to deeper layers [10]. Rapid resistive heating at 90 W may achieve effective superficial modification of the slow pathway while reducing the risk of injury to the compact AV node [11].

The rationale for selecting the specific combination of high power (90 W) and short duration in vHPSD ablation lies in its capacity to generate wide, shallow lesions while minimizing conductive heat transmission to deeper tissue layers. The optimal contact force in the left atrium typically ranges from 10–25 g, with an inter-lesion distance of 5–6 mm [12,13]. A recent preclinical study by Nakagawa et al. demonstrated that tissue temperatures and lesion dimensions (depth, diameter, and volume) were lowest for RF applications at 90 W/4 s, followed by 50 W/10 s, and highest for 30 W/30 s [14]. Increasing contact force was shown to significantly increase lesion depth across all ablation modalities.

These findings provide a strong rationale for employing smaller and more precise lesions in the slow pathway region. In some patients, this target zone, located along the anterior margin of the coronary sinus near the tricuspid valve, is narrow and highly mobile, rendering it unfavorable for prolonged applications. The vHPSD approach, by using a 4-s application time, may therefore reduce the likelihood of catheter instability. In our case, the average contact force was 10 g, with adequate catheter stability maintained throughout the procedure.

Given that arrhythmic paroxysms occurred on a weekly basis, the six-month follow-up period provides convincing evidence of efficacy. However, in view of the potential for late-onset AV conduction disturbances previously reported, continued long-term monitoring remains warranted to fully assess procedural safety.

In this case, transient second-degree AV block was likely due to reversible perinodal edema rather than irreversible damage, as full recovery of AV conduction occurred within hours. This supports the hypothesis of limited lesion depth. The short duration of energy application also contributes to a reduction in overall procedural time.

Although vHPSD ablation has been investigated in atrial fibrillation and ventricular tachycardia, its use for slow pathway modification in AVNRT has not previously been reported. This approach may be especially advantageous in redo procedures or refractory cases. However, the potential for AV nodal injury at such high power requires cautious application, meticulous monitoring, and further validation.

Future prospective studies are warranted to define lesion characteristics, procedural safety, and long-term efficacy of this novel vHPSD strategy in AVNRT ablation.

## 4. Conclusions

Very high-power, short-duration ablation at 90 W for 4 s may represent a feasible and potentially effective alternative for the treatment of AVNRT refractory to conventional ablation. This first-in-human experience demonstrates the technical feasibility of this approach and establishes a foundation for future clinical investigation.

## Data Availability

All data available upon reasonable request from the authors due to privacy reasons.
